# Longitudinal multi-omic changes in the transcriptome and proteome of peripheral blood cells after a 4 Gy total body radiation dose to Rhesus macaques

**DOI:** 10.1186/s12864-023-09230-7

**Published:** 2023-03-21

**Authors:** Shanaz A. Ghandhi, Shad R. Morton, Igor Shuryak, Younghyun Lee, Rajesh K. Soni, Jay R. Perrier, James Bakke, Janet Gahagan, Kim Bujold, Simon Authier, Sally A. Amundson, David J. Brenner, Denise Nishita, Polly Chang, Helen C. Turner

**Affiliations:** 1 Center for Radiological Research, Columbia University Irving Medical Center, 630, W 168th street, VC11-237, New York, NY 10032 USA; 2 Proteomics and Macromolecular Crystallography Shared Resource, Herbert Irving Comprehensive Cancer Center, NY New York, 10032 USA; 3 Biosciences Division, SRI, 333 Ravenswood Avenue, Menlo Park, CA 94025 USA; 4 Charles River Laboratory, 445 Armand-Grappier Blvd, (QC) H7V 4B3 Laval, Canada

**Keywords:** Radiation, Hematological acute radiation syndrome, Non-human primates, Radiation dosimetry, Hematology, Transcriptomics, Proteomics, Blood, Biofluid, Cytogenetics

## Abstract

**Background:**

Non-human primates, such as Rhesus macaques, are a powerful model for studies of the cellular and physiological effects of radiation, development of radiation biodosimetry, and for understanding the impact of radiation on human health. Here, we study the effects of 4 Gy total body irradiation (TBI) at the molecular level out to 28 days and at the cytogenetic level out to 56 days after exposure. We combine the global transcriptomic and proteomic responses in peripheral whole blood to assess the impact of acute TBI exposure at extended times post irradiation.

**Results:**

The overall mRNA response in the first week reflects a strong inflammatory reaction, infection response with neutrophil and platelet activation. At 1 week, cell cycle arrest and re-entry processes were enriched among mRNA changes, oncogene-induced senescence and MAPK signaling among the proteome changes. Influenza life cycle and infection pathways initiated earlier in mRNA and are reflected among the proteomic changes during the first week. Transcription factor proteins SRC, TGFβ and NFATC2 were immediately induced at 1 day after irradiation with increased transcriptional activity as predicted by mRNA changes persisting up to 1 week. Cell counts revealed a mild / moderate hematopoietic acute radiation syndrome (H-ARS) reaction to irradiation with expected lymphopenia, neutropenia and thrombocytopenia that resolved within 30 days. Measurements of micronuclei per binucleated cell levels in cytokinesis-blocked T-lymphocytes remained high in the range 0.27–0.33 up to 28 days and declined to 0.1 by day 56.

**Conclusions:**

Overall, we show that the TBI 4 Gy dose in NHPs induces many cellular changes that persist up to 1 month after exposure, consistent with damage, death, and repopulation of blood cells.

**Supplementary Information:**

The online version contains supplementary material available at 10.1186/s12864-023-09230-7.

## Background

Exposure to radiation and its subsequent adverse health effects may impact many individuals, such as in the case of a dirty bomb explosion in a crowded city or a radiation accident [1]. The subsequent lethal effects of radiation for a few, but also the potential exposure to the larger proportion of the population, have driven the need to identify and validate radiation biomarkers over a range of physical and biological measurements, in both simple and complex exposure scenarios [2–6]. According to the FDA’s Animal Rule [7, 8], large animal models, such as non-human primates, are the closest to simulating the human response to radiation, as NHPs have similar tissue and organ structure with the added advantage of enabling sequential sampling during a relatively long lifespan [9]. Genetically, NHP are similar to humans, with 93% DNA sequence similarity [10, 11]. After exposure to radiation, NHPs display similar clinical, histological, and physiological symptoms to those in humans, which makes them most suitable for whole- and partial-body irradiation dose response studies [12, 13]. Many groups have extended the testing of candidate radiation biomarkers from different tissues/biofluids using the Rhesus macaque, *Macaca mulatta* and other Macaca species [12, 14–16]; and baboons [13, 17, 18]. In humans, most studies have focused on biofluids such as whole blood, serum or isolated PBMCs, but biofluids are also analyzed in in vivo models such as mice [19–22], dogs [23], and pigs [24]. This study was designed to elucidate the hematopoietic-acute radiation syndrome (H-ARS) response to a sub-lethal radiation dose in NHP to simulate the response of healthy humans to a similar dose. The lethal dose for Rhesus macaques is ~ 6.6 Gy (LD_50/60_) [24, 25] compared to the LD_50_ of 4.5 Gy in human [26].

A number of -omics studies from this NHP cohort and others are available in the literature including metabolomics and proteomics in urine and serum and organ systems [27–30]. A comprehensive tandem mass spectrometric (TMT) analysis and immunoassay based approach revealed radiation biomarkers up to day 7 in NHP [31] that were comparable to those in human radiotherapy patients [32]. Candidate miRNA biomarkers in plasma have also been identified in NHP up to 7 days after acute doses [33].

Cytogenetic analysis of radiation-induced chromosomal aberrations in peripheral blood lymphocytes (e.g., dicentrics, translocations, micronuclei) are widely accepted as biomarkers of exposure to ionizing radiation to assess and estimate the DNA damage [34]. The advantage of these cytogenetic endpoints is their long biological half-life making them suitable for retrospective biodosimetry analysis after radiation exposure [35]. In our earlier work, we showed the persistent expression of micronuclei in peripheral blood T-lymphocytes of Rhesus macaques, 8 months after a single 10 Gy whole thorax lung irradiation dose [16].

The objective of the current study was to identify and compare the induction and persistence of different radiation -omics and cytogenetic responses in NHP peripheral blood in vivo after a 4 Gy TBI exposure. We also compared complete blood cell counts (CBC) with Acute Radiation Syndrome (ARS) severity scores as reported in King et al., which are METREPOL (MEdical TREatment ProtocOLs for Radiation Accident Victims) human scores modified for H-ARS in NHP [5, 36, 37]. We used network analysis to connect multi-level information from mRNA and proteins in the time series and also report long-term cytogenetic DNA damage effects and recovery. The goal of our report is to link global genomic and proteomic responses, cytogenetic and blood sub-population changes indicative of acute ARS to understand the mechanisms underpinning H-ARS.

## Results

### Animal body weight and clinical observations

**Fig. 1:**
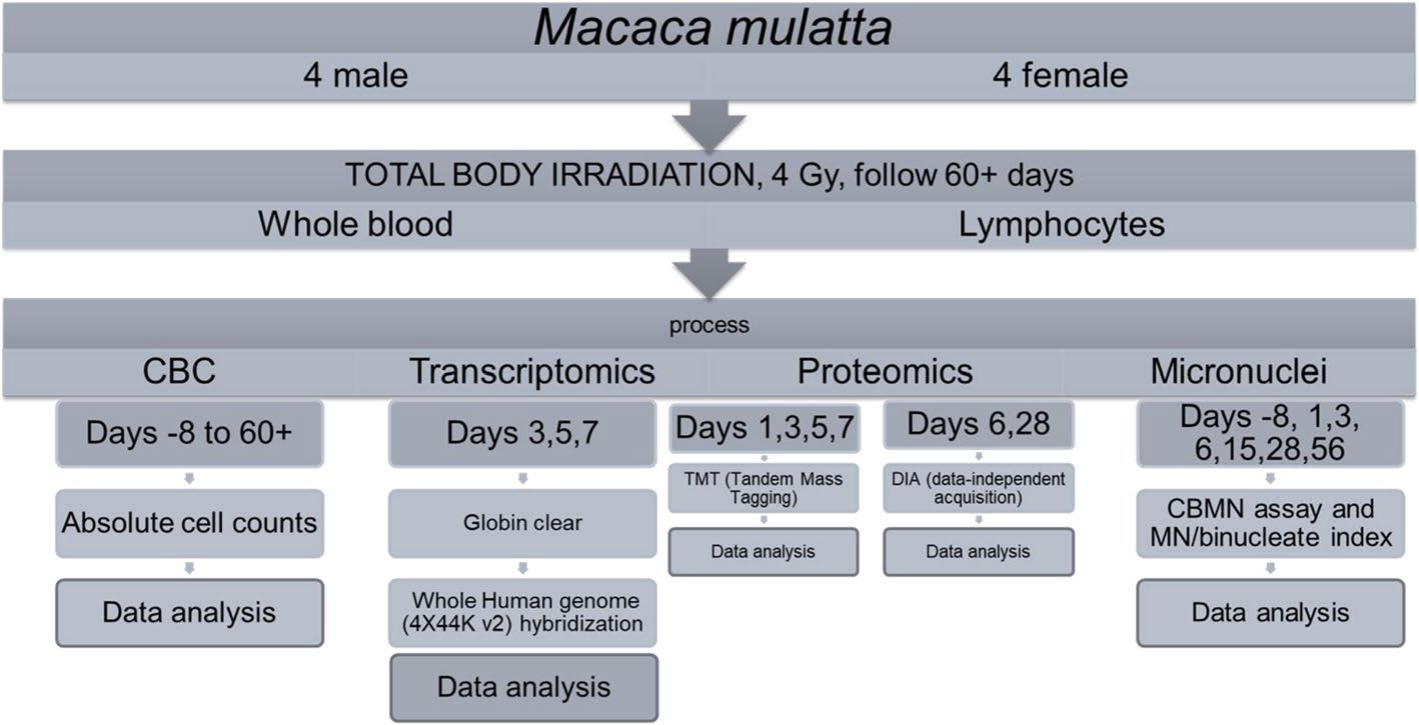
Study summary and scheme of sampling in the time course. Statistical cut offs for each endpoint are mentioned in the Materials and Methods section

Mean body weight decreased in males following irradiation up to day 56; however, two males showed slightly decreased body weight up to day 7 while the other two males showed decreased body weights up to day 56. Similarly, a slight decrease in mean body weight was noted in females following irradiation up to day 7 (less than 2%). Most animals had sporadic days of decreased food appetence noted. One animal presented with severely decreased food appetence and signs of emesis mostly between days 12 and 20. Other clinical signs were considered incidental since they occurred across groups including the sham irradiated group, showed no relationship to irradiation in incidence or severity and were considered incidental. Following irradiation, serial sampling and processing was performed following the scheme in Fig. 1 (Supplementary Table 1 for details).

### Hematology

**Fig. 2:**
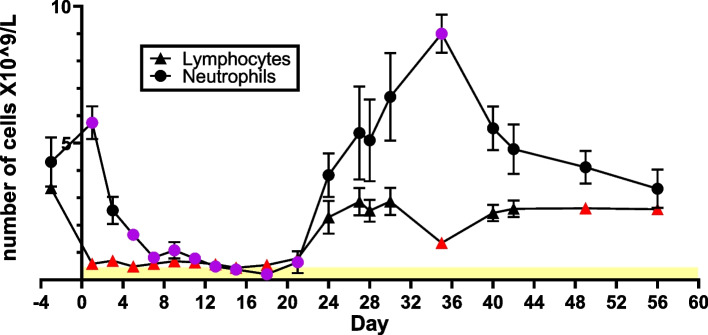
Mean absolute count (× 10^9^/L) of lymphocytes and neutrophils in NHP peripheral blood up to day 56 after TBI. Statistical analysis showed that measurements at certain time points were significantly different from pre-irradiated levels (paired t-tests, *p*-values < 0.05) and are indicated as colored points (red for lymphocytes, purple for neutrophils) in the graph. The yellow region at the bottom of the graph corresponds to severe lymphopenia and neutropenia [33]

Following whole body irradiation, hematology parameters of white blood cells (WBCs), neutrophils, lymphocytes, platelets, and red blood cells (RBCs) decreased, providing evidence of radiation-induced myelosuppression with pancytopenia in irradiated animals while hematology parameters were unaffected in sham animals throughout the study up to 56 days. Figure 2 shows the mean data for lymphocyte and neutrophil counts for all eight irradiated NHPs. The results show that overall leukocyte counts decreased immediately following irradiation, leading to significant cell loss/large drop in cell numbers observed at 3 days post irradiation that continued to gradually decrease and reach nadir values by day 18, then returned to baseline equivalent values by day 27. Lymphocyte counts declined drastically within 24 h post-radiation (-84.6% from pre-radiation levels), reaching the nadir within 7 days that lasted up to day 15 when levels began to recover close to baseline values by day 27. The severe lymphocytopenia initiated on day 1 was counterbalanced by an increase in neutrophil count. The marked increase in the mean neutrophil count noted on day 1, was ascribed to a stress formula with neutrophil demargination. Afterwards, neutrophil levels decreased rapidly reaching the lowest absolute neutrophil count by day 18, which recovered to baseline comparable values around day 24. Levels continued to increase up to day 35, followed by a slow decline to pre-irradiation levels by day 56. The mean duration of neutropenia was from days 13 to 28.

**Fig. 3:**
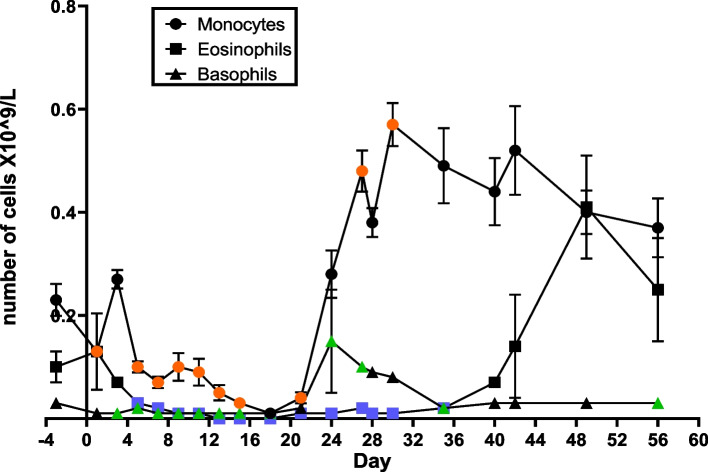
Mean absolute count (× 10^9^/L) of monocytes, eosinophils, and basophils in NHP peripheral blood after TBI. Statistical analysis showed that measurements at certain time points were significantly different from pre-irradiated levels (paired t-tests, *p*-values < 0.05) and are indicated as colored points (orange for monocytes, blue for eosinophils and green for basophils) in the graph

The mean absolute count of monocytes, eosinophils, and basophils in the peripheral NHP blood samples are shown in Fig. 3. Although these leukocyte subtypes show a different response at the early time points after radiation exposure, all three cell types reach the nadir at around day 18. Notably, monocyte cell levels begin to rapidly increase on day 21, peaking on day 30 at levels almost double those of the pre-irradiation, whereas eosinophils show a late doubling on day 49. Monocytosis continues for a couple more weeks, highlighting an apparent immune response to inflammation and infection. *P*-values for the mean relative and absolute hematology cell counts can be found in Supplementary Table 2.

**Fig. 4:**
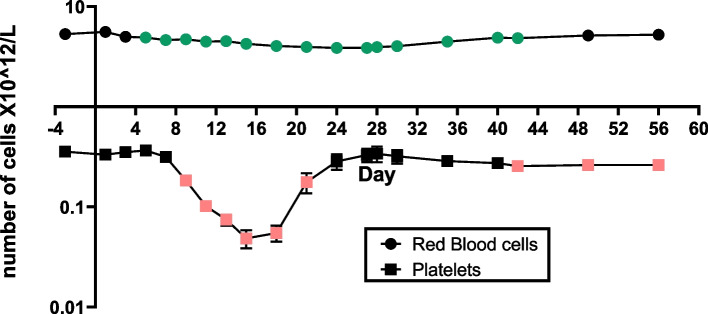
Mean absolute count (× 10^12^/L) of red blood cells and platelets in NHP peripheral blood after TBI. Statistical analysis showed that measurements at certain time points were significantly different from pre-irradiated levels (paired t-tests, *p*-values < 0.05) and are indicated as colored points (green for red blood cells and pink for platelets) in the graph

Due to their longer half-life compared to other blood cells, platelet counts started to decrease on day 9, reaching a nadir on day 15, followed by recovery close to baseline values on day 28. Erythrocytes also present relatively long half-lives; however, since there were extensive blood collections, the RBC levels started to decrease on day 3, with values that remained lower from days 15 to 30 and returned to baseline values around day 40, Fig. 4. The mean reticulocyte count began to decline the day after irradiation reaching nadir on day 5 and was followed by a compensatory increase up to approximately day 13, which is characteristic of the regenerative response to radiation-induced anemia. A second moderate decline in reticulocyte count was observed between days 15 and 21, which may have resulted from iron sequestration typically observed with inflammation (i.e., anemia or chronic inflammation), followed by an approximate threefold increase from baseline levels. Mean relative and absolute hematology cell counts measured in all the irradiated animals over the study period is presented in Supplementary Table 2.

### Microarray analysis

Results of the global transcriptomic changes after irradiation with 4 Gy are summarized side-by-side with proteomics results in Table 1. Overlap between differentially expressed genes and proteins throughout the time course are also summarized in the Venn diagrams (Supplementary data Fig. 1). mRNA changes in whole blood cells affected a total of 5435 genes with 1148 genes in common at all time points. The overlap between time points was roughly half of all differentially regulated genes and there were 2133 genes in common between days 3 and 5, and 1869 between days 5 and 7. This suggests that even though a large number of genes were affected over the time course, these time points represent a continuum of radiation gene expression responses with specific signaling and mRNA changes observed at the time points selected.

**Table 1:** Summary of molecular changes in blood of NHP after total body irradiation with 4 Gy

**Day**	**Transcripts** (% up)	**Proteins** (% up)
1	–	135 (55%)
3	3693 (46%)	86 (34%)
5	4296 (49%)	–
6	–	1978 (35%)^a^
7	3123 (38%)	781 (70%)
28	–	630 (21%)^a^

^a^2^nd^ batch of samples sent for proteomics analysis using DIA methodology

**Fig. 5:**
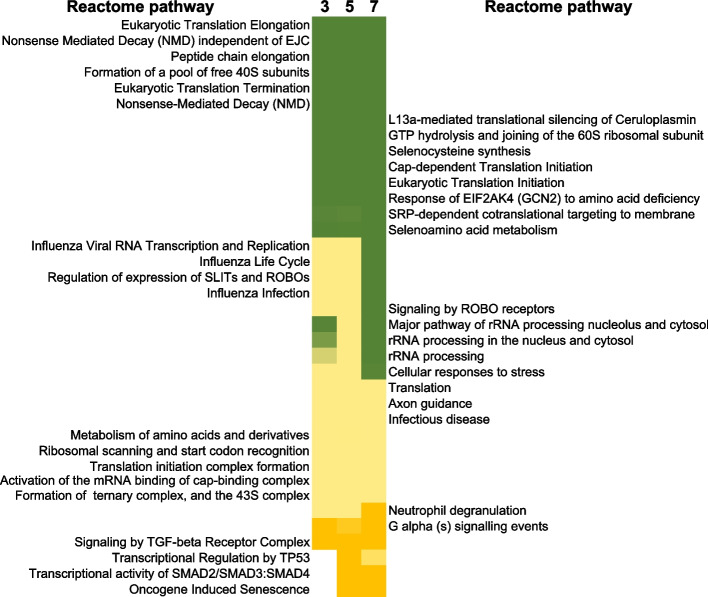
REACTOME analysis of mRNA changes up to 1 week. Using PANTHER gene expression analysis tool, selecting for Macaca mulatta organism, using the statistical overrepresentation test (Reactome pathways) and Macaca mulatta reference whole genome, then selecting Fisher’s exact test and Bonferroni correction for multiple testing, the REACTOME categories that passed the corrected Bonferroni *p*-value cut off of 0.05 are shown below. Next, we merged the list and used VLOOKUP in Microsoft excel to sort the Bonferroni *p*-values in ascending order at the 3 time points as shown. The color scale indicated the range of Bonferroni *p*-values from deep green (10^–36^) to yellow (10^–2^) for these pathways. Overlapping processes such as eukaryotic translation, influenza life cycle and infection, rRNA processing were affected at all time points up to 1 week at the mRNA level. Processes related to Oncogene induced senescence and SMAD regulation of transcriptional activity appeared only at day 5 to 7

To understand the biological response playing out after irradiation, we further analyzed these gene lists using ontology tools in PANTHER database. The results of our analyses are listed (in Supplementary data table 3) and more focused REACTOME pathway analyses are shown in Fig. 5. The REACTOME database builds pathways based on prior knowledge from the literature and biological interactions between molecules, DNA, RNA, protein, and others [38]. It is a curated database of pathways and reactions in human biology and can be used for multi-omics analyses [39]. Reactions are considered as pathway ‘steps’ such as binding, activation, translocation, degradation, and classical biochemical events involving a catalyst. Information in the database is collated and curated and cross-referenced by expert biologists and editorial staff. We used the inferred orthologous reactions that are available for human data in place of *Macaca mulatta* data.

**Fig. 6:**
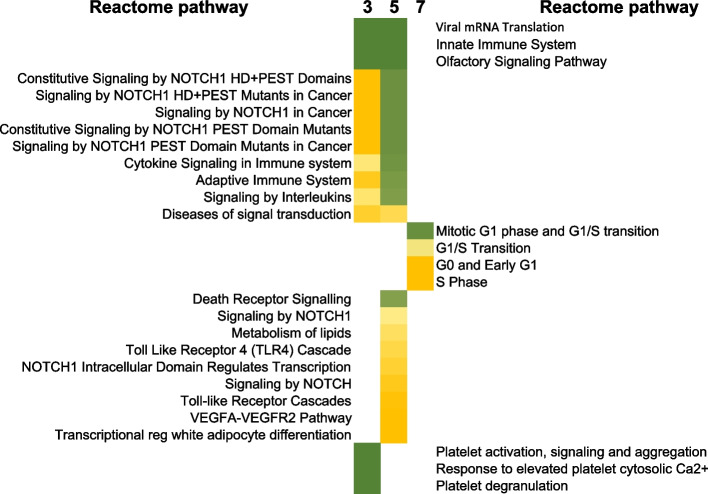
Reactome analysis of mRNA changes up to 1 week, pathways that were affected at specific times up to 1 week after radiation. Data analysis was performed as described for Fig. 4, however here we chose to sort processes that only were significant at the early time points, day 3 and 5, and then those that were only significant at only one time point. The color scale indicated the range of Bonferroni *p*-values from deep green (10^–36^) to yellow (10^–2^) for these pathways

Sorting the results of pathway analyses by time point and grouping significant pathways by day within the time course, resulted in 2 groups of signaling changes after radiation: 1) pathways affected consistently up to 1 week, shown in Fig. 5, and 2) pathways that were affected at 1 or 2 specific time points after radiation, listed in Fig. 6. There were no signaling pathways that were significantly changed at the earliest day 3 and latest day 7 (not at day 5), time points in these analyses. Of note among the pathways that were significantly affected at all time points after radiation were broad processes related to stress (Bonferroni *p*-value < 10^–14^), infectious disease (Bonferroni *p*-value < 10^–10^), processes related to ribosomal changes and translation of proteins (Bonferroni *p*-value < 10^–8^) and neutrophil degranulation (Bonferroni *p*-value < 10^–5^) at all time points. Among disease categories, influenza viral RNA transcription and regulation and life cycle were also significant (Bonferroni *p*-value < 10^–18^) at all times in the first week after irradiation. More focused pathways such as Gαsignaling events (Bonferroni *p*-value < 10^–3^) and TGFbeta receptor signaling (Bonferroni *p*-value < 10^–2^) were also persistently affected at all points in the gene expression measurements of this time course.

Overall, the changes that persisted at all time points within the first week after irradiation (group 1) are broad cellular changes, affecting neutrophil granulation, protein translation and ribosomal machinery changes. A more detailed investigation of the changes that were significant at one or 2 time points in the time course, paints a more complete picture of the evolving signaling over 1 week.

Starting with TP53 signaling at day 5 (Bonferroni *p*-value < 10^–3^) and continuing to day 7, the expected DNA damage response involving the ubiquitous transcriptional regulator TP53 was significant (Fig. 5). This was not observed at day 3. Further, transcription of SMADs 2,3 and 4 also began only at day 5 (Bonferroni *p*-value < 10^–2^), which could be a result of the earlier TGFbeta signaling seen at day 3. Surprisingly, oncogene induced senescence pathway was also significant starting at day 5 (Bonferroni *p*-value < 10^–2^) and persisting to 1 week. Other early response pathways that were significantly only up to day 5 involved NOTCH signaling (Bonferroni *p*-value < 10^–2^), innate and adaptive immune system (Bonferroni *p*-value < 10^–2^) and interleukin and cytokine signaling (Bonferroni *p*-value < 10^–3^). These broad processes were not significantly affected at day 7 in the time course.

Pathways that were significant only at 1 time point showed dramatically different biology, with platelet degranulation (Bonferroni *p*-value 10^–6^), platelet activation, signaling and aggregation (Bonferroni *p*-value < 10^–8^) and olfactory signaling (Bonferroni *p*-value < 10^–9^) at day 3. Day 5 specific signaling processes were death receptor signaling (Bonferroni *p*-value < 10^–4^), metabolism of lipids (Bonferroni *p*-value < 10^–3^) and NOTCH signaling (Bonferroni *p*-value < 10^–2^). Most interestingly, the processes and pathways significant only at day 7 were those related to mitosis, G1/S transition, G0 and early G1 (Bonferroni *p*-value < 10^–2^), indicating a strong cell cycle phase response.

### Proteomics analysis

**Fig. 7:**
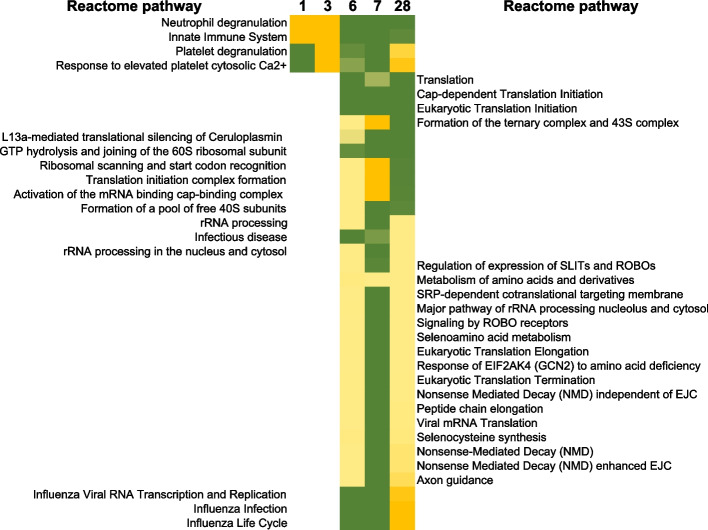
REACTOME analysis of protein changes up to 28 days after TBI. Using PANTHER gene expression analysis tool, selecting for Macaca mulatta organism, using the statistical overrepresentation test (Reactome pathways) and Macaca mulatta reference whole genome, then selecting Fisher’s exact test and Bonferroni correction for multiple testing, the REACTOME categories that passed the corrected Bonferroni *p*-value cut off of 0.05 are shown below. Next, we merged the list and used VLOOKUP in Microsoft excel to sort the Bonferroni *p*-values in ascending order at the 3 time points as shown. The color scale indicated the range of Bonferroni *p*-values from deep green (10^–36^) to yellow (10^–2^) for these pathways. Processes related to immune system, platelet and neutrophil degranulation were significant at all times at the protein level. The lower part of the heatmap are processes that were significant starting at day 6 and then also at the latest 28-day time point

**Fig. 8:**
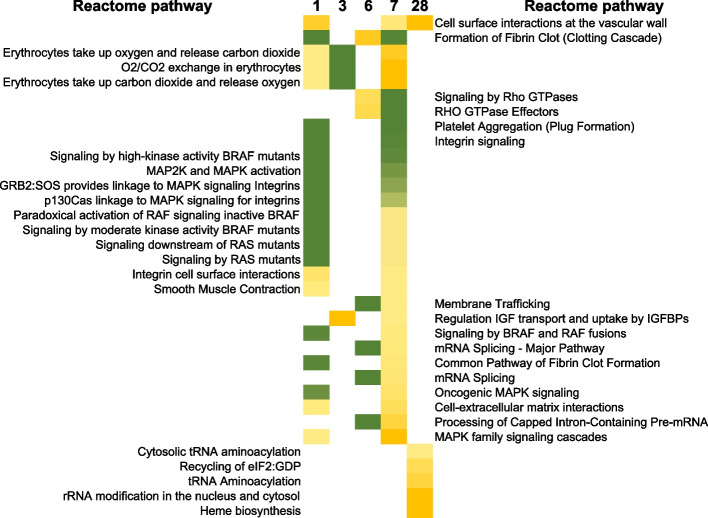
REACTOME analysis of protein changes up to 28 days after TBI. Data analysis was performed as described for Fig. 6, however here we chose to sort processes that were mostly significant within 1 week after TBI. The color scale indicated the range of Bonferroni *p*-values from deep green (10^–36^) to yellow (10^–2^) for these pathways

Results of the global proteomic changes after irradiation with 4 Gy are summarized in Table 1. Protein changes in PBMCs affected a total of 2763 proteins with 2 proteins in common at all time points (Supplementary Fig. 1). The lack of overlap in proteins across the 4-week time course was likely driven by low yields at day 3 with only 86 proteins detected as differentially regulated. The 6-day time point in this analysis gave the most unique set of proteins with 1379 proteins not changed at any other point in the time course, roughly half of all proteins changed. In order to understand the biological response playing out after irradiation, we further analyzed these lists of proteins using ontology tools in PANTHER database. The results of our analyses are listed (in Supplementary data table 4) and more focused REACTOME pathway analyses are shown in Figs. 7 and 8 below.

As explained previously with transcript analysis, the REACTOME database allows us to identify and sort pathways by time point, and we grouped significant pathways by day within the time course, resulting in 4 groups of signaling changes after radiation: 1) pathways affected consistently up to 4 weeks; 2) pathways that were unaffected at the early time points days 1 and 3, but started responding at day 6 and then at day 28; 3) pathways that were affected at multiple time points within the first 1 week; and finally group 4) that were affected only at 1 specific time point after radiation, listed in Supplementary data table 5.

Group 1 consisted of very broad categories of biological changes in neutrophil degranulation (Bonferroni *p*-value < 10^–2^), platelet degranulation (Bonferroni *p*-value < 10^–2^), platelet response to Ca^2+^ (Bonferroni *p*-value < 10^–2^), Fig. 7.

Group 2, consisted of pathways that only started responding at day 6 onwards consistently to day 28, and these were cellular activities involving protein translation (Bonferroni *p*-value < 10^–12^), rRNA processing (Bonferroni *p*-value < 10^–6^), amino acid metabolism (Bonferroni *p*-value < 10^–6^) and eukaryotic translation elongation and termination (Bonferroni *p*-value < 10^–5^). Disease specific pathways related to influenza infection and life cycle (Bonferroni *p*-value < 10^–3^) and broader cellular response to stress (Bonferroni *p*-value < 10^–3^) were also significant within this time period starting at ~ 1 week. Next in group 3, were pathways that were activated at multiple time points within the first week, these were erythrocyte uptake of O_2_ and release of CO_2_ (Bonferroni *p*-value < 10^–2^), platelet aggregation (Bonferroni *p*-value < 10^–8^), MAPK and MAP2K signaling (Bonferroni *p*-value < 10^–6^) and fibrin clot formation (Bonferroni *p*-value < 10^–4^). These pathways were not significant at the latest 28-day time point, Fig. 8.

Finally, group 4, that consists of pathways that appear to be time specific starting at day 1, were RAF/MAP kinase signaling (Bonferroni *p*-value < 10^–5^), MAPK1/MAPK3 signaling (*p*-value < 10^–5^) and FLT3 signaling (Bonferroni *p*-value < 10^–5^). At day 3, there were only two pathways, TLR signaling (Bonferroni *p*-value < 10^–3^) and amyloid fiber signaling (Bonferroni *p*-value < 10^–2^). The lack of information at this time may be due to few proteins significantly changed at this time point. At day 6, the number of unique processes were higher in number due to the increased number of differentially regulated proteins, with mRNA splicing (Bonferroni *p*-value < 10^–10^), mature mRNA transport (Bonferroni *p*-value < 10^–14^), mitochondrial translation (*p*-value < 10^–8^), and SUMOylation of proteins (Bonferroni *p*-value < 10^–6^) as significant cellular functions affected at this time. At the same day 6, a different set of pathways related to HIV life cycle (Bonferroni *p*-value < 10^–9^), viral RNA synthesis (Bonferroni *p*-value < 10^–8^), rev protein import (Bonferroni *p*-value < 10^–7^), HCMV early events (Bonferroni *p*-value < 10^–6^) and anti-viral IFN stimulated genes (Bonferroni *p*-value < 10^–6^) were significantly changed. G2/M transition (Bonferroni *p*-value < 10^–3^), metaphase and anaphase proteins (Bonferroni *p*-value < 10^–2^) and RNA polymerase II transcription (Bonferroni *p*-value < 10^–2^) were significant. Surprisingly, pathways only significant at day 7 were different from day 6 with receptor tyrosine kinases (Bonferroni *p*-value < 10^–8^), phagocytic cup formation (Bonferroni *p*-value < 10^–4^), VEGFA-VEGFR2 interactions (Bonferroni *p*-value < 10^–3^), and GLUT4 translocation to the plasma membrane (Bonferroni *p*-value < 10^–2^) affected at the 1-week point. Finally, pathways that were only affected at 4 weeks after irradiation, were related to tRNA aminoacylation (Bonferroni *p*-value < 10^–5^), rRNA modification (Bonferroni *p*-value < 10^–2^) and heme biosynthesis (Bonferroni *p*-value < 10^–2^) (see full list with Bonferroni *p*-values in Supplementary data file 4).

### Merging mRNA and protein results

**Fig. 9:**
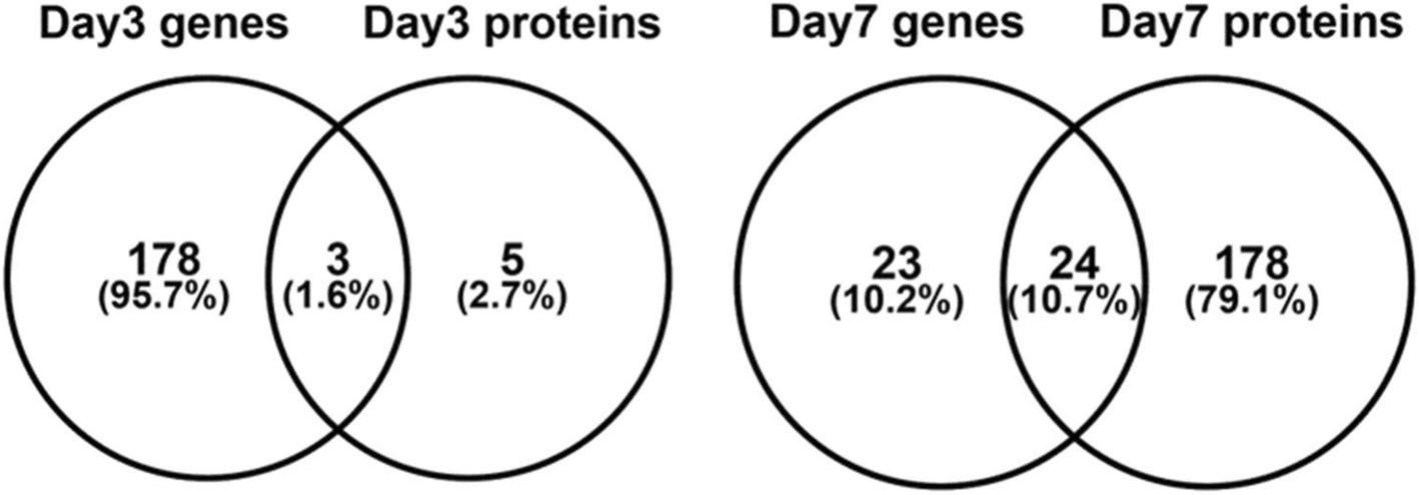
Overlap of enriched biological processes using gene ontology from the significantly regulated transcripts and protein at days 3 and 7 for which we had multi-omic datasets. There were some common biological processes at both time points but also unique processes suggesting differences in kinetics of the two types of molecular targets measured at the same time point

We first looked at overlap between mRNA and protein measurements at 3 and 7 days after irradiation for which we had both mRNA and protein -omic data. At 3 days there were 20 mRNAs and proteins in common (between 3693 mRNA and 86 proteins), and 105 at 7 days (between 3123 mRNA and 781 proteins). These common molecules are listed in Supplementary data file 5 as a Venn diagram and list of common molecules. We also analyzed these lists of mRNAs and proteins using PANTHER gene ontology based on biological processes and enrichment using Bonferroni correction for multiple tests [40]. Figure 9 shows the overlap of significant biological processes at 3 and 7 days at both mRNA and protein levels, with 3 in common at 3 days and 24 in common at 7 days after irradiation. These common processes were related to immune system, response to bacteria and external stimuli at the 3-day point. The common processes at 7 days were related to cellular and nitrogen metabolic processes and stress response at both mRNA and protein levels, suggesting a shift in the molecular landscape between the time points.

We also attempted to correlate mRNA and proteins with time across the 1-week time course, and this analysis revealed that few genes such as *FAU, HBB, RPS14, FBL, EPB42, BLVRB, SNCA, RPS24, SLC16A1* and *AQP1* were seen to correlate negatively with time (< 7 days, *R* < -0.5); and *PTMS* and *AHSP* both mRNA and proteins also were consistently downregulated in the first one week (*R* < -0.5). Attempts to predict upstream regulators of transcription of these mRNA and correlations with protein changes revealed no common factors (Supplementary table 6, second tab).

**Fig. 10:**
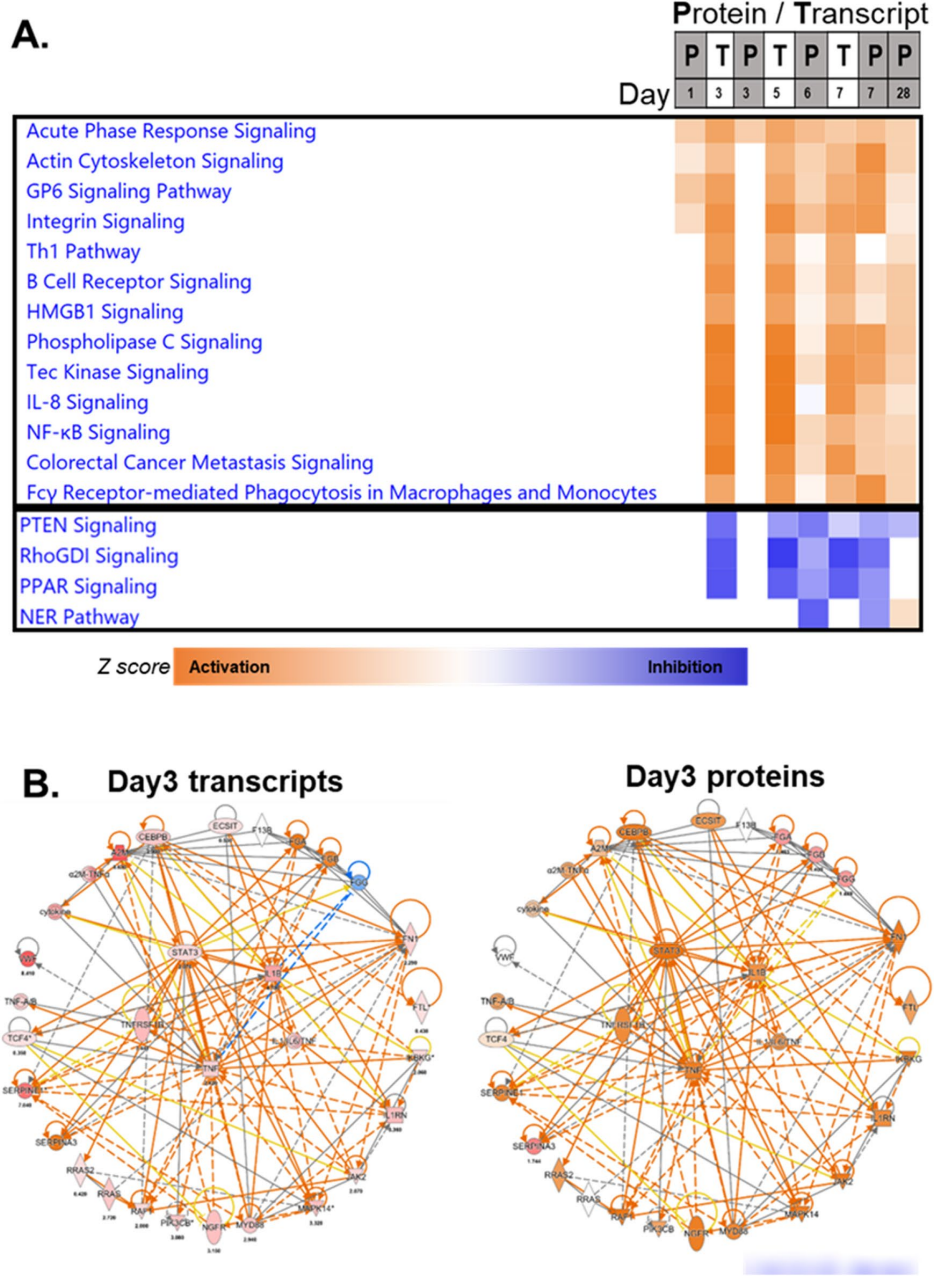
Ingenuity pathway analysis (IPA) of all transcriptional and proteomics changes in chronological order. **A** Heatmap of top IPA pathways that were activated (orange cells in heatmap on the right-hand side represent positive z scores) or inhibited (blue cells in heatmap on the right-hand side represent negative z scores) in both -omics analysis and across the 28-day time course are shown (range between -4.796 to 6.091). Acute phase response signaling pathway was the only biological response that was predicted to be activated at all times and at both transcript and protein levels. **B** Network analysis of molecules in acute phase response signaling network. In the center of the radial circle are known radiation response transcription regulators and molecules (nodes) on the circumference of the circle are predicted targets. The colors of the nodes depict up regulation (shades of red) or no change (blank node) at the transcript (left) and protein (right) level at 3 days after irradiation. Orange and blue nodes indicate no change at the transcript or protein level itself, however, the color indicates potential activation (orange) or inhibition (blue) of the regulator. Many of the up-regulated transcripts in the network on the left, are also ‘activated’ at the protein level on day 3 as shown in the network on the right

Using network and pathway analysis tools in Ingenuity Pathway Analysis explorer program [41] we aligned the data from mRNA and protein -omic analysis by day and proceeded to analyze activation and inhibition of well-known pathways across the 1-month time course (in a total of 8 gene and protein lists of differentially expressed molecules). The top activated and inhibited pathways by z-score are shown in Fig. 10A. The top pathway across all times regardless of mRNA or protein activation was the acute phase response signaling pathway. The range of z-scores for this pathway was between 2 and 4, suggesting strong activation of this response. The pathway with strongest inhibition across the time course was PTEN signaling, with z-scores between -1 and -3. Other signaling pathways that were significantly activated at the earliest times between 1 and 3 days were actin cytoskeleton signaling, glycoprotein 6 (collagen receptor) signaling and integrin signaling pathways. Among the strongly inhibited pathways were RhoGDI, PPAR and NER signaling pathways. A closer and more detailed view of the network of transcriptional and protein changes at the earliest common time point for activation of the top activated pathway: acute phase response signaling at day 3 is shown in Fig. 10B, where the central players in the network of molecules are shown in the center of the radial network. These are STAT3, IL1B, TNFRSF1B, TNF family and IL1/IL6/TNF family of activation molecules. The network on the left is overlaid with mRNA level changes, shades of red indicating increased mRNA levels compared with unirradiated controls. As can be seen in the transcripts network at 3 days, a large number of downstream transcriptional targets are up regulated in addition to the central activators in this pathway. On the right, in the protein network, with the same outline structure, the predicted activation levels can be seen in the center players (indicated by orange-colored nodes), and these are surrounded by the same molecules as the network on the left, except that the nodes are colored using the protein expression information. On the circumference most of the nodes are activated with some of these also expressed at higher protein levels in irradiated cells compared with controls. Overall, this network analysis displays a more granular view of some of the central players in the acute phase response, that contribute to the strong activation scores for this pathway at day 3.

**Fig. 11:**
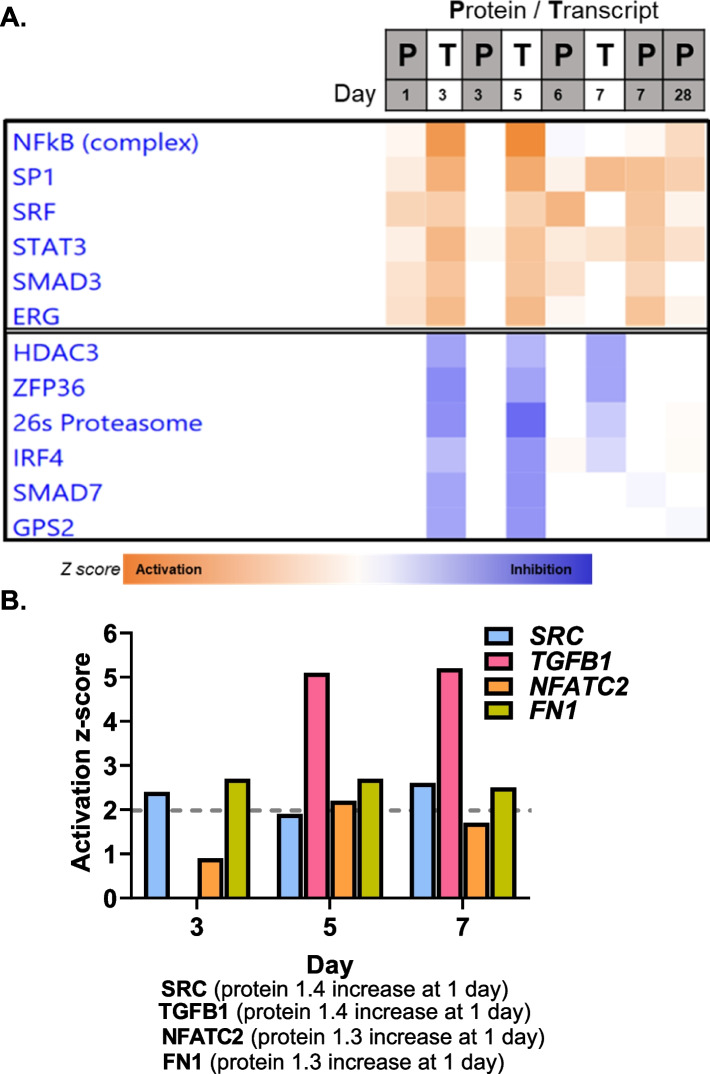
Ingenuity pathway analysis of upstream transcriptional regulators of -omic changes in chronological order. **A** Heatmap of top IPA upstream transcriptional regulators that were activated (orange cells in the heatmap, representing positive z-scores) or inhibited (blue cells in the heatmap, representing negative z-scores) in both transcriptomic and proteomic measurement are shown. Expectedly, NFκB and STAT3 are potentially activated after radiation exposure, with inhibition of HDAC3 and the 26s proteasome. **B** Differential protein changes at day 1 (proteomics) compared with predicted transcriptional activity as measured by z-scores (range between -6.102 to 9.304) at following days (transcriptomic predictions) are shown. Regulators such as SRC, TGFbeta, NFATC2 and FN1 are increased at the protein level as well as predicted to be activated up to 1 week later by transcriptional changes

We also used the -omic information to better understand potential activation/inhibition of transcriptional regulation between protein regulators and mRNA targets. Again, here we aligned all 8 lists of significantly regulated mRNA and proteins across the time course and analyzed potential upstream regulation for these molecules. Prediction of the activation state of these upstream regulators is shown in Fig. 11A, as a heatmap with top activated regulators such as NFK®B SP1, and STAT3, which are known to be involved in radiation response. Inhibited regulators are also shown in the lower block in Fig. 11A, such as HDAC3, IRF4 and ZFP36. We queried the results of the upstream analysis approach to identify proteins that showed significant changes after irradiation at the earliest time point (1 day after irradiation), that were also predicted to be activated. We then looked for signs of this activity in the observed mRNA changes up to 1 week. Seen in Fig. 11B, are z-scores of predicted transcriptional activations for 4 proteins (SRC, TGFB, NFATC2 and FN1), all up regulated at the protein level at day 1. The results show that at least for some of the earliest protein level changes (at 1 day after irradiation), there were correlating transcriptional activations reflected at later times in differentially regulated mRNA as would be expected. The difference in z-score profiles over time may reflect the different kinetics of activity of these proteins.

### Micronuclei Frequency in peripheral blood lymphocytes

**Fig. 12:**
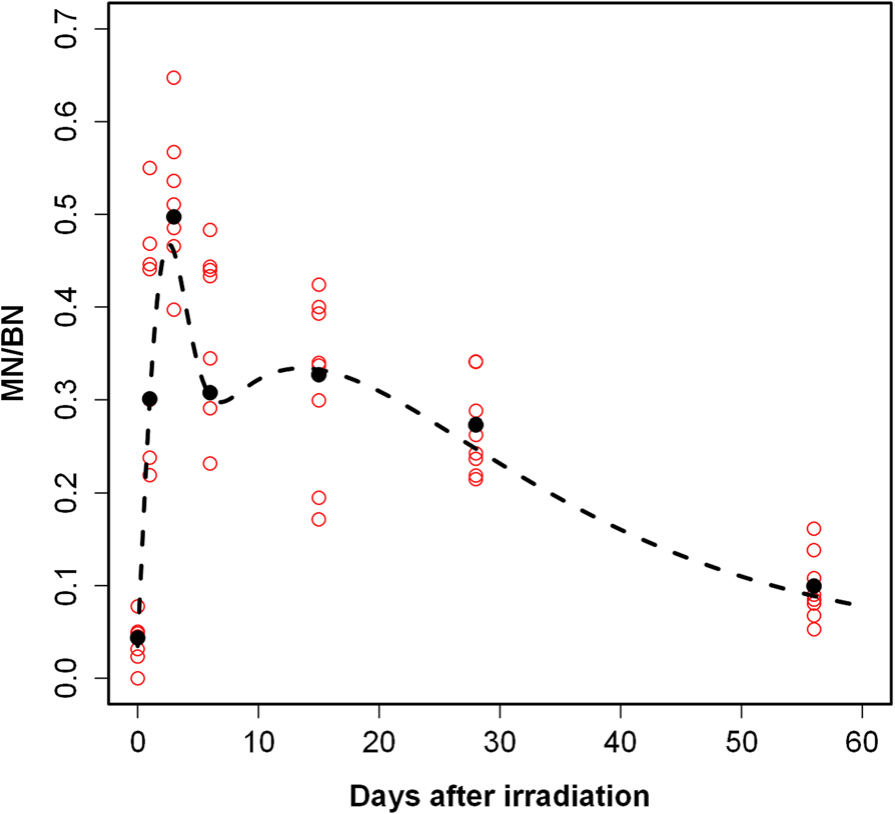
Time dependence of micronuclei in NHP lymphocytes after 4 Gy TBI. The MN/BN for each individual animal up to day 56 were fitted using a nonlinear regression model (black dashed line derived from Eq. 1). The five best-fit parameter values shown in Supplementary Table 8. The global mean for the observed MN/BN levels at each specific time point is include

Figure 12 shows the time course for micronuclei per binucleate (MN/BN) levels in the peripheral blood NHP lymphocytes measured up to 56 days after 4 Gy TBI exposure. The number of BN cells and MN levels for each individual NHP across the study time points can be found in Supplementary Table 7. The data show that the largest MN/BN values ( 0.497) were observed at day 3, followed by a rapid dip at day 6 ( 0.308). Overall, MN/BN yields remained elevated in the range 0.273–0.327 up to 28 days and declined to 0.1 MN/BN by day 56. The data were fitted (dashed line in Fig. 12) using a non-linear time course model (Eq. 1) based on the best-fit parameter values shown in Supplementary Table 7. A comparison of the MN/BN proportions at day -8 (pre-irradiation) vs day 56 showed that for five of the irradiated animals (2001, 2002, 2003 and 2501, 2503) there was a persistent expression of MN yields that was significantly (*p* < 0.05) elevated above non-irradiated baseline levels. In all cases, there was a significant difference (*p* < 0.005) in blood samples collected with heparin or ETDA, where seven of the NHPs (except male 2001) showed increased MN/BN levels in heparin-treated samples.

Overall, the total observed binucleate cell counts in the day 1 samples were 50% less than those measured in the day 3 samples. We speculate that this could be due to a stress-like signaling response in vivo that has reduced the effectiveness of mitogen stimulation in vitro. The reduced number of total BN cells on day 15 that significantly increases by day 28 correlates with the hematology recovery of the blood lymphocyte population. A comparison of observed and model-predicted (fitted) MN/BN values is provided in Supplementary Table 7. It indicates a good correlation between the observed and predicted mean MN/BN values. A mixed-effects version of the model, with random effects for the baseline parameter C, was not very different from the fixed effects version (Eq. 1). The random effects standard deviation for parameter C was very small, only 7.34 × 10^–7^, suggesting that inter-individual variability in baseline MN/BN values did not have a big impact on model predictions. More complicated mixed effects model structures with random effects for other parameters (e.g., Kprod, P) could not be fitted reliably to the available data set. These results suggest that the simple fixed effects model (Eq. 1) had reasonable performance in describing the main data trends over time after irradiation (Fig. 1).

## Discussion

This study was part of a larger multi-site study performed by SRI/CRL, from which 2 groups at Center for Radiological Research at Columbia University Irving Medical Center (NY) received whole blood. Samples/biofluids were distributed to different sites to perform various endpoint analyses to analyze the long-term effects of total body irradiation, extending to weeks after 4 Gy TBI exposure, some of which are already available in the literature [27, 42–44]. Long term studies in NHP can have a significant advantage over human studies that are performed ex vivo and at shorter time points, extensively reviewed in [19]. The dose chosen here represents a higher dose level of exposure, with symptoms of hematopoietic acute radiation syndrome (H-ARS) and concurrent pancytopenia [45]. In this study, shared samples at time points extending to 28 days were distributed to different sites to perform various endpoint analyses. We restricted the mRNA measurements to within 1 week, protein measurements started day 1 through day 28 and cytogenetic analyses were extended to day 56. Although samples were collected from both sexes, the study was under-powered to differentiate sex-specific differences and we considered the results of both sexes together. Hematology was performed at shorter and regular intervals throughout the time course to capture the damaging effects of radiation and recovery, up to 2 months reported here.

Hematology results showed that the expected lymphopenia and thrombocytopenia associated with HARS was observed early in the first week after exposure with days of nadir and day of recovery for lymphocytes (nadir: day 15, recovery: day 27), neutrophils (nadir: day 18, recovery: day 27), monocytes (nadir: day 18, recovery: day 24), basophil (nadir: day 18, recovery: day 24), eosinophil (nadir: day 15, recovery: day 42), RBC (nadir: day 27, recovery: day 56) and platelets (nadir: day 15, recovery: day 28) (Figs. 2, 3 and 4). A comparison of CBC absolute counts with a scoring system based on the human METREPOL action [5, 46] but modified for NHP, showed that the 4 Gy TBI effect was between mild (score 1, based on hemoglobin levels at > 20 days between 80–109 g/dL and HCT (hematocrit) > 20 days between 0.27–0.32%) and moderate (score 2, based on platelet levels at > 8 days at < 180 X 10^9^/L, neutrophil levels between 10 – 21 days at < 1.2 X 10^9^/L and lymphocyte levels between 1 -14 days at 0.45-1 X 10^9^/L, see Supplementary table 2) [36]. Comparison of cell count recovery trends with Rhesus macaques exposed to 6 Gy TBI were comparable, with neutropenia and lymphopenia resolving within 30 days [47].

We also measured two molecular endpoints using -omics approaches, global transcriptional and protein changes using microarrays and mass spectrometry, respectively. Using shared samples collected within the first week after exposure, we compared mRNA and proteomic results after irradiation. Overall, the sublethal dose of 4 Gy TBI to Macaca induced many transcriptional and protein changes in blood cells up to four weeks. At the earlier time points a few thousand transcriptional changes were observed, comparable with total body irradiation in the mouse model [20, 48, 49], in spite of cell depletion of lymphocytes and platelets in blood. Trends in protein changes were fewer in number with very few protein changes at day 3, suggesting correlation with loss of lymphocytes and cellular material. At later times within the first week, the number of protein changes increased to 1978, however, as we detail in the methods, these measurements were performed using a different more advanced platform (DIA), which may contribute to this difference (Table 1). For both transcripts and proteins, the distribution between up and down regulated molecules were close indicating that even though there were significant cell losses at these times, there was no overall trend towards down-regulation of transcripts or proteins driven by cell losses. About a third (1148) of all DEG were changed at all time points in the first 7 days, while very few proteins were found in common across the 28-day time course, mostly due to very few measured changes in proteins at 3 days.

Separate pathway analysis of transcript and protein results provides a dynamic picture of cellular changes and potential physiology, and with concurrent measurements of cell counts and DNA damage we can potentially infer correlations between results from different endpoints. First, we considered transcriptional and protein changes separately because there were only two coincidental measurements, on days 3 and 7 to compare directly. Transcriptional changes across the 1-week time course were consistent with observed cellular changes with enrichment of processes related to coagulation, neutrophil degranulation, and cell division (Fig. 5). Other processes that were inferred from the transcriptional changes such as influenza RNA metabolism and infection were consistently significant at all times measured up to 7 days, which may be related to a general inflammatory response. At later times, days 5 and 7, transcriptional changes in TP53, SMADs (TGFbeta signaling) and even oncogene-induced senescence suggest activation of DNA damage response and repair, after the initial cell loss. Remaining cells may enter temporary growth arrest as they repair damage from the acute exposure that may continue past the first week. Biological processes significantly changed at 7 days, were in NOTCH signaling and signal transduction by interleukins consistent with an early inflammatory response reflected in mRNA changes (*NOTCH1* mRNA was 2.24-fold up regulated (5 days), *NOTCH2* mRNA 3.64-fold (5 days) and 3.66-fold (7 days) and *NOTCH4* mRNA 2.4-fold (3 days) up after irradiation) that may be associated with later protein/cellular responses in the NOTCH pathway. Notch signaling generally affects radiosensitivity of cancer cells but may also impact normal signaling after TBI [50–52].

Processes that were unique to the time points were interesting as they reveal the changing transcriptional profile/functions of blood cells over time (Fig. 6). Interestingly, at day 3 platelet activation, aggregation and degranulation were enriched functions but not seen at days 5 and 7. At day 7, cell cycle processes including G0 to G1, G1 to S phase and S phase are enriched, indicating that there is a concerted repair and re-entry into cell cycle after damage at this time.

Time course proteomic responses were analyzed similarly, with biological enrichment of neutrophil and platelet degranulation consistent across 4 weeks after TBI, indicating release of granules after activation of these cells. Processes that started within at 6 days and were also seen at 28 days, were housekeeping eukaryotic translation initiation and elongation, rRNA processing and nonsense mediated decay of RNA. Additionally, starting slightly later than mRNA changes, influenza infection and life cycle processes were enriched starting at day 6 and continuing to 28 days (Fig. 7). Processes that were significantly affected within the first week after irradiation but not extending out to 28 days included formation of fibrin clots, platelet (plug formation) and erythrocyte-oxygen metabolism, which may correlate with the migration of platelets and depletion from peripheral blood as observed by hematological changes (Fig. 8). At the final time point, protein changes related to heme biosynthesis were enriched, indicating a correlation with the beginning of renewal of RBC (Fig. 4).

Next, we compared responses of both mRNA and proteins at the two coincidental time points, days 3 and 7, where we found similar functions at both molecular levels that were related to immune response, infection, metabolism, and overall stress response (Fig. 9). Shared biology in the mRNA and protein changes only confirmed what we knew from previous biological analyses. A more complete and comprehensive experimental design for multi-omics analysis would include closer time points with more matched times for molecular measurements, however, due to the scarcity of NHP and limitations of serial bleeding the animals after irradiation, we were only able to capture shared samples from a few time points and only 2 shared points. As a way to analyze the -omics data from two different sources, we merged the data regardless of whether they were mRNA or protein and analyzed them as a time course consisting of days 1, 3, 3, 5, 5, 6, 7, 7 and 28 data.

Using network and pathways analysis in IPA, we used the merged molecule lists (independent of type) and performed a core analysis of pathways and regulators of gene expression (Fig. 10A). Acute phase response to radiation was revealed as the top pathway activated at all times in the 28-day time course (positive z-scores). A more detailed view of members of this pathway revealed IL1B, IL6, TNF and STAT3 as central players in regulating this signaling response that is well conserved across mammalian species [53]. The acute phase response pathway is a typical response to inflammatory stress and has many similarities of symptoms with acute radiation sickness, such as fever, leukopenia, and malaise [53–55]. In particular, C reactive protein (CRP) levels are found to be increased at day 28 in the blood, Serum amyloid A (SAA4) up + 5-fold at day 6, Haptoglobin (HP) up + 3.6-fold at day 28, FGA  at -11.6-fold down regulated at day 6, but Fibrinogen A and B (FGA and FGB) up + 1.7 and + 1.6-fold at day 28. Ceruloplasmin (CP) is + 1.4 at day 1 and + 2.6 at day 6. We compared our pathway results with those from another study of the transcriptomic response after 6.5 Gy TBI in Rhesus macaques [56] and found that the acute phase response pathway was significantly changed after irradiation in the blood (-log_10_ *p*-value 1.9 at day 1 in survivors, 1.6 in non-survivors at day 1; and 2.4 in non-survivors at day 2) similar to our results after 4 Gy TBI. Other pathways that showed similar significant response after irradiation with 6.5 Gy were HMGB1 and PPAR signaling pathways (Fig. 10A).

We delved further into the time course response looking for upstream regulators (independent of molecule type), which revealed NFKB, STAT3 and SMAD3 as mostly activated and HDAC3 and IRF7 as inhibited among the protein and transcript profiles. Among activated transcriptional regulators that belonged to cytokines, IL8 was found to be activated among the mRNA changes consistently up to 1 week and for proteins up to 28 days (Fig. 10A). In a different study on 6.5 Gy TBI exposed NHP, IL3 was found to be strongly dysregulated at 24 h, which was not found after 4 Gy in our study [56]. These results alongside the biological analysis suggest that there is a rapid response after 4 Gy TBI involving cellular and DNA damage, with concurrent molecular changes that evolve over time. A concerted DNA damage stress and immune response persist in the first week, implicating viral life cycle response and infection pathways. The acute phase response suggested from the molecular data and elevated MN levels up to 28 days with cell count changes implies that there is a persistent response to the whole-body dose that does not subside to pre-irradiation levels even up to 1 month after exposure.

Based on these observations and time points of data acquisition in the early 1-week period after irradiation, we investigated whether combining information about proteins and potential activation of proteins predicted by mRNA changes might suggest primary activators of signaling. We found that there were proteins such as SRC, TGFB, NFATC2 and fibronectin1 (FN1) that were significantly up regulated at protein levels and also predicted to be activated at early times (day 1 for proteins and days 3 to 5 for mRNA) (Fig. 11A). These are strong candidate proteins for the immediate response and may drive activation of transcription of target genes throughout the time course.

In this work, we also developed a high-throughput NHP-CBMN assay protocol to measure MN frequency in small volumes of peripheral whole blood. Overall, the data show relatively high levels of MN/BN in the range 0.273–0.327 up to 28 days after 4 Gy TBI exposure which declined to 0.1 MN/BN by day 56 (Fig. 12). The persistent expression of MN in peripheral blood lymphocytes weeks after radiation exposure measured here, highlights the important use of MN biomarker as an in-vivo dosimeter, which when corrected for time after radiation, can be used to accurately reconstruct absorbed dose. Previous studies using the NHP model for biodosimetry have shown that the frequency of mature T-cells carrying unstable (dicentrics, micronuclei) chromosomal aberrations decreases with time after radiation exposure [16, 57, 58]; reflecting their turnover (cell death and re-population) in the circulating blood. More recently, the advanced development of high-throughput CBMN [58] and dicentric assay systems [35] have accurately reconstructed dose in human and NHP blood exposed ex vivo. In addition, the CytoRADx assay platform developed by ASELL, has also been used to accurately reconstruct dose in NHPs exposed in vivo to TBI doses 0–8 Gy, 1–28 days post-irradiation [58].

The changing biology after 4 Gy TBI with 10 Gy WTLI [16] using NHP blood is comparable, with early cellular changes in platelets and neutrophils and later immune response related to virus and infection. DNA damage and cell cycle response was observed early after WTLI but was not apparent in early TBI samples. Gene expression patterns following both irradiation modes were similar in direction. Overall, these cellular/cytogenetic results with concurrent molecular changes in the blood cells create a fuller picture of the changes after total body irradiation. Recently, we have developed a micro-array based gene expression signature from ex-vivo irradiated human blood that can reconstruct acute doses in a continuous manner between 0 and 8 Gy [59]. We tested whether the Macaca homologues of the human signature genes from mRNA data in this work (and an unpublished data set from 7.4 Gy TBI acute dose exposed Rhesus macaques) both at day 3 also correlate with dose. Initial comparisons indicate good correlation with dose for the NHP homologs of human dose response genes (Supplementary data table 8) at day 3, however, additional data would be needed to establish usefulness of these genes in NHP for extended times and doses.

## Conclusions

The challenge with such multi-omics approaches in large animal models that lend themselves to serial tests, remains in the limitations of timing of sampling, and this study elucidates the difficulty clearly. Sharing of samples was limited to only 3 days for transcriptomics with only two time points in common with the proteomics measurements. Future studies should be carefully designed, powered for both sexes, and samples collected at as many matched and regularly spaced time points as possible to get a better understanding of the molecular interplay and signaling in a time course [60]. Additional information of protein half-lives can be used to connect mRNA kinetics to protein changes in the same system [61], however, this may not elucidate the question of drivers of radiation response as potential drug targets using trans-omics [62]. One of the great advantages of this type of study, however, is in following the physiology of individual animals up to 2 + years after exposure, which may yield some insight into early molecular changes that impact later outcomes.

## Materials and Methods

### Animal care, irradiations, and sample sharing overview

This study was conducted according to the Standard Operating Procedures of CiToxLAB North America (now Charles River, USA). All work was conducted in CiToxLAB North America’s animal research facilities, which are fully accredited by the Association for Assessment and Accreditation of Laboratory Animal Care (AAALAC, number 001056) and the study is reported in accordance with ARRIVE guidelines. Rhesus macaques (*Macaca mulatta*, Chinese origin) at average age 6 y and average weight 8.3 kg were irradiated following the guidelines of the protocol. Animal maintenance, veterinary care and irradiation protocols are described in detail in the metabolomics study on the same NHP cohort published previously [27].

At the time of irradiation, the age of the animals ranged from 5 to 10 years with body weights that ranged from 6.3 to 21.9 kg for males and from 4.6 to 6.2 kg for females. Four male and 4 female NHPs were total body irradiated with 4 Gy at a dose rate of 0.6 Gy/min using a ^60^Co γ source over the course of ~ 7 min (1/2 exposure anterior–posterior position, 1/2 exposure posterior-anterior position using a Theratron 1000, Best Theratronics, Ottawa, ON, CA). LD_50/60_ for this animal model is ~ 6.7 Gy and the sub-lethal 4 Gy dose was chosen to enable the continuous sampling and longitudinal monitoring of biological, physiological, and molecular changes over a longer span of time i.e., 60 days and longer. In this study we collected samples for mRNA measurements up to 1 week, protein measurements up to 28 days, and cytogenetics up to 56 days after irradiation. Pre-irradiation samples were taken between 3–8 days before the day of irradiation (Supplementary Table 1 has details of animal numbers used in each time point).

During this study, animals were observed daily for mortality checks with detailed examinations that were performed pre-irradiation and at 1, 3, 5 and 7 days after irradiation; then weekly thereafter (particular attention was paid to the incidence of febrile neutropenia and infections) up to 60 days. Clinical signs were observed twice as well as diarrhea assessment. Body temperature was recorded prior to irradiation and daily after irradiation, up to 7 days, then weekly up to 60 days. Food appetence was monitored daily. Body weights were recorded at pre-irradiation, at 1, 3, 5 and 7 days and then every 3 days until 30 days, and then weekly thereafter up to 60 days. At the end of the study animals were observed for at least 92 days prior to transfer to Wake Forest (North Carolina) for long term housing.

### Hematology

Clinical pathology evaluations were performed (ADVIA 120 Hematology Analyzer, Siemens, USA) on all animals at the following time points: 3 occasions pre-irradiation (-12, -8, and -3 days), on days 1, 3, 5, 7, 9, 11, 13, 15 after irradiation, and then on the same occasions as subsequent investigator blood collections up to 60 days (Fig. 1 shows the scheme of treatment and sampling for different endpoints). Food was not removed overnight prior to blood collections. Statistical analysis showed that measurements at certain time points were significantly different from pre-irradiated levels (paired t-tests, *p*-values < 0.05).

### Blood collection for RNA isolation

1.0 mL whole blood was collected into pre-labeled PAXgene RNA tubes containing 4.0 mL PAXgene solution (catalog number 762165, Becton Dickinson, New Jersey), using a syringe at 3 days prior to irradiation and then post irradiation at 1, 3, 5 and 7 days (Fig. 1, transcriptomics endpoint arm). The tube was tightly closed and then gently mixed by inverting 8–10 times immediately after addition of blood. Samples were stored upright at room temperature for at least 2 h. Tubes were then initially frozen at -20 °C for at least 24 h and then transferred to -80 °C until shipment on dry ice to Columbia University Irving Medical Center, New York, NY.

### RNA isolation and processing using microarrays

Globin-cleared RNA samples were hybridized to Agilent Whole Genome Human 4X44 v2 (catalog number: G4845A) microarrays using the manufacturer’s protocol, based on inter-species cross reactivity microarray tests between *H. sapiens* and *Macaca mulatta* [63, 64]. Feature extraction of the images was performed using Agilent’s FE version 10.7 software and data were imported into BRB ArrayTools software version 4.6.0. The gene expression dataset was deposited in the NCBI GEO database under the accession number GSE113478. Data were processed using standard procedures (described in [16, 65], normalized to the median array, class comparisons were performed at each time point to determine lists of differentially expressed genes after radiation compared with pre-irradiated controls, using the Benjamini–Hochberg correction, FDRs < 0.005.

### Global quantitative proteomics of NHP PBMCs

Fresh whole blood samples (~ 1.0 ml) were collected in a 2 mL BD vacutainer tube with K_2_EDTA (catalog number 367841; Becton Dickinson; Franklin Lakes, NJ) and inverted 8–10 times (Fig. 1, proteomics endpoint arm). Samples were shipped the same day directly to Columbia University Irving Medical Center, New York, NY, using a Crēdo Cube™ shipper (Peli BioThermal, MN). The inclusion of a temperature data logger (catalog number EL-USB-1; Lascar Electronics; Erie, PA) with each blood shipment confirmed that samples were shipped between the desired range of 16-29ºC.

Peripheral blood mononuclear cells (PBMCs) were isolated from the NHP blood samples by Ficoll density gradient. Peripheral whole blood samples (~ 900 µl) were diluted in 5 mL of RPMI medium (Gibco, Waltham, MA) and layered over 6 mL of Histopaque1083 (Sigma-Aldrich, St. Louis, MO). Samples were spun at 1220 rpm for 45 min at room temperature, and the isolated PBMCs were transferred to a 15 mL conical tube and washed twice with 10 mL of PBS. Cell pellets were frozen and stored at − 80 °C until use. Samples were submitted in two batches to the Proteomics and Macromolecular Crystallography Shared Resource, Herbert Irving Comprehensive Cancer Center, New York: batch A included samples collected from all the pre-irradiated animals (day -8; *n* = 10), and the irradiated animals (days 1, 3 and 7; *n* = 8); batch B included blood samples collected from the two unirradiated animals across 2 times points (days 6 and 28; *n* = 8) and the irradiated animals (days 6 and 28; *n* = 8).

For proteomic sample preparation and analysis, TMT (Tandem Mass Tagging)-based proteomic quantification method was performed on the batch A samples and the next-generation proteomics platform, DIA (data-independent acquisition) was used for batch B. A full description of the TMT proteomics approach is described in our earlier work [66]. Details of the DIA-based proteomics method are described here [67, 68]. Briefly, tissues were resuspended in SDC lysis buffer (1% SDC, 10 mM TCEP, 40 mM CAA and 100 mM TrisHCl pH 8.5) and boiled for 10 min at 60 °C, mixed at 1500 rpm to denature and reduce and alkylate cysteine, followed by sonication in a water bath, then cooled down to room temperature [67]. Protein concentration was estimated by BCA measurement and 20 µg were further processed for overnight digestion by adding LysC and trypsin in a 1:50 ratio (µg of enzyme to µg of protein) at 37 °C and 1500 rpm. Peptides were acidified by adding 1% TFA, vortexed, and subjected to polymer sorbent StageTips exchange micropipette tips clean-up via SDB-RPS. Twenty µg of peptides were loaded on two 14-gauge StageTips plugs. Peptides were washed two times with 200 µL 1% TFA 99% ethyl acetate followed 200 µL 0.2% TFA/5%ACN in centrifuge at 3000 rpm, followed by elution with 60 µL of 1% Ammonia, 50% ACN into Eppendorf tubes and dried at 60 °C in a speed vacuum centrifuge. Peptides were resuspended in 10 µL of 3% acetonitrile/0.1% formic acid and injected on a Thermo Scientific™ Orbitrap Fusion™ Tribrid™ mass spectrometer with DIA method for peptide MS/MS analysis [68]. The UltiMate 3000 UHPLC system (Thermo Scientific) and EASY-Spray PepMap RSLC C18 50 cm × 75 μ ID column (Thermofisher Scientific) coupled with Orbitrap Fusion (Thermofisher) were used to separate fractioned peptides with a 5–30% acetonitrile gradient in 0.1% formic acid over 70 min at a flow rate of 250 nL/min. After each gradient, the column was washed with 90% buffer B for 5 min and re-equilibrated with 98% buffer A (0.1% formic acid, 100% HPLC-grade water) for 40 min. Survey scans of peptide precursors were performed from 350–1200 m*/z* at 120 K FWHM resolution (at 200 m*/z*) with a 1 × 10^6^ ion count target and a maximum injection time of 60 ms. The instrument was set to run in top speed mode with 3 s cycles for the survey and the MS/MS scans. After a survey scan, 26 m/z DIA segments will be acquired at from 200–2000 m*/z* at 60 K FWHM resolution (at 200 m*/z*) with a 1 × 10^6^ ion count target and a maximum injection time of 118 ms. HCD fragmentation was applied with 27% collision energy and resulting fragments were detected using the rapid scan rate in the Orbitrap. The spectra were recorded in profile mode.

### Proteomic data analysis

*Batch A*: Proteome Discoverer software (version 2.2) was used to search the acquired MS/MS data against Macaca protein database (2017) downloaded from the UniProt website (uniprot.org) and to generate TMT ratios. Positive identification was set at 5% peptide false discovery rate (FDR). Also, at least 1 unique peptide must be identified per protein. Duplicated protein identifications from database were removed. TMT ratios (each channel/common reference) were calculated by PD 2.2 and normalized by total peptide amount. Qlucore Omics Explorer 3.3 & Prism 6 Software were used to perform correlation and statistical analysis. log_2_ transformation and KNN imputation was used for missing values.

*Batch B*: DIA data were analyzed with direct DIA 2.0 (Deep learning augmented spectrum-centric DIA analysis) in Spectronaut Pulsar X, a mass spectrometer vendor-independent software from Biognosys (Switzerland). Spectronaut was set up to search with the reference Macaca proteome database (2021) downloaded from UniProt. The default settings were used for targeted analysis of DIA data in Spectronaut except the decoy generation was set to “mutated”. The false discovery rate (FDR) was estimated with the mProphet approach and set to 1% at peptide precursor level and at 1% at the protein level. Results obtained from Spectronaut were further analyzed using the Spectronaut statistical package. Significantly changed protein abundance was determined by un-paired t-test (comparing irradiated to unirradiated animals) with a threshold for significance of *p* < 0.05 (permutation-based FDR correction) and 0.58 log_2_FC. The mass spectrometry proteomics data have been deposited to the ProteomeXchange Consortium via the PRIDE partner repository [69] with the dataset identifier PXD037807.

### Gene ontology, network, and pathway analysis

We used PANTHER database gene expression tools [70, 71] to analyze the differentially expressed gene lists generated from the microarray and proteomic datasets with the human annotations database and REACTOME pathways and biological processes categories [38]. Multiple testing was performed based on Bonferroni corrections and adjusted *P* values (< 0.05) are reported [72]. We also analyzed sets of up- and down-regulated genes separately and looked for common biological processes among these genes. We also uploaded the lists of genes to Ingenuity Pathway Analysis® Software (IPA from Ingenuity®: http://www.ingenuity.com) and performed prediction analysis for upstream regulators and enriched canonical pathways. This method identifies potential upstream regulators and ranks them by statistical significance by providing information about the direction of activation of regulatory protein based on the downstream gene targets from the gene list. The program provides a z score derived from the number of target genes, their relative expression, and the type of relationship between the regulator and target genes (either activation or inhibition) from the published literature. We also compared gene lists using the Venny tool [73].

### Cytokinesis-block micronucleus (CBMN) assay

We adapted our high-throughput and miniaturized platform designed for the human CMBN assay [74, 75] and previously published cell culture protocols [16, 76, 77] to develop the whole blood NHP-CBMN assay protocol used here. Whole blood samples (50–100 µL) were pipetted into 1.4 ml 2D-barcoded tubes (Matrix™ storage tubes; Thermofisher Scientific Inc., Waltham, MA) within a 96-tube rack and washed with 1 ml of RPMI-1640 medium (Life Technologies, Grand Island, NY) containing heparin (1.58 units/ml; sourced from a BD vacutainer). Given that the fresh blood samples were collected in K_2_EDTA vacutainers for the proteomic arm of the study, based on our own previous experience, we washed the cells with an RPMI-heparin solution to prevent clotting during the assay culture time. After washing, the blood samples were cultured with RPMI-1640 supplemented with 15% heat-inactivated fetal bovine serum (HI-FBS; Life Technologies), concanavalin A (10 ug/mL; Sigma-Aldrich), and 1% penicillin/streptomycin (10,000 U/mL; Life Technologies). Cells were cultured at 37 °C in a humidified atmosphere with 5% CO_2_ for 68 h, after which time cytochalasin B (Sigma-Aldrich) was added to the culture medium for an additional 26 h at a final concentration of 6 μg/mL. At the end of the assay time, the plates were spun, and the pelleted cells were treated for 8 min with hypotonic solution (0.075 M KCl, Sigma-Aldrich) and fixed with methanol/glacial acetic acid (ratio 4:1) and stored at 4 °C. A small drop of fixed cell suspension was dropped onto a clean glass microscope slide and air-dried. The nuclei were counter-stained with DAPI Vectashield® mounting medium (Vector Laboratories, Inc., Burlingame, CA). [74, 78]. Scoring and analysis of micronuclei per binucleated cells were quantified using Zeiss fluorescence microscope (Axioplan 2; Carl Zeiss MicroImaging Inc., Thornwood, NY) × 10 air objective lens and Metafer MSearch platform.

For statistical analysis, the two-proportions Z-Test (implemented using the prop.test function in *R* 4.2.0 software) was used to compare MN/BN values for each animal for day 56 vs day 0 samples. Separate comparisons were performed for day 56 samples collected using the EDTA vs Heparin blood collection protocols. Correction of *p*-values for multiple testing was performed by the Bonferroni method.

We analyzed the time course of MN/BN values over the time range of day 0 to day 56. Based on mechanistic reasoning, we assumed that elevation of the MN/BN value above the baseline is determined by competing processes of production and removal/decay over time. We considered 4 candidate model formalisms with different power dependences on time and compared their performances using the Akaike information criterion (AIC). All model variants were fitted using nonlinear weighted regression with weights proportional to 1/SEM^2^, where SEM are standard errors of the mean for the MN/BN samples. The weights were normalized so that the mean weight across all samples was set to 1. Fitting was performed using the *nls* function in *R* 4.2.0 software.

The preferred model structure with the best AIC score is described by the following equation (Eq. 1), where Day is the day since irradiation, C is the baseline MN/BN value before irradiation (Day -8 in the experiments, but assigned to 0 for regression modeling purposes), Kprod is a term for production of the micronuclei, Kdec_1_ and Kdec_2_ are linear and quadratic exponential decay (repair and/or removal of micro-nucleated cells) rates over time, and P is the fraction of decay using Kdec_1_:

1 $$\frac{{\varvec{M}}{\varvec{N}}}{{\varvec{B}}{\varvec{N}}}=\mathbf{C}+\mathbf{K}\mathbf{p}\mathbf{r}\mathbf{o}\mathbf{d}\times \mathbf{D}\mathbf{a}\mathbf{y}\times (\mathbf{P}\times \mathbf{e}\mathbf{x}\mathbf{p}(-{\mathbf{K}\mathbf{d}\mathbf{e}\mathbf{c}}_{1}\times \mathbf{D}\mathbf{a}\mathbf{y})+(1-\mathbf{P})\times \mathbf{e}\mathbf{x}\mathbf{p}(-{\mathbf{K}\mathbf{d}\mathbf{e}\mathbf{c}}_{2}\times {{\varvec{D}}{\varvec{a}}{\varvec{y}}}^{2})$$

A mixed effects version of this model, where random effects were added to parameter C to represent baseline variability by donor, was also investigated, using the nlme *R* function. An absolute performance metric that was calculated for each variant was residual standard error, which measures the standard deviation of the residuals. It was relatively low (0.0366) for the preferred model shown in Eq. 1.

## Supplementary Information

**Additional file 1: Supplementary data table 1. **Summary of samples processed in each endpoint.

**Additional file 2: Supplementary data table 2.** Hematology detailed table.

**Additional file 3: Supplementary data table 3.** Differentially expressed genes by day.

**Additional file 4: Supplementary data table 4.** Differentially expressed protein lists.

**Additional file 5: Supplementary data table 5.** Gene ontology on gene and protein lists.

**Additional file 6: Supplementary data table 6.** IPA analysis.

**Additional file 7: Supplementary data table 7.** MN data tables.

**Additional file 8: Supplementary data table 8.** Correlation of gene expression response between NHP and Human.

**Additional file 9: Supplementary Figure 1.** Overlap of differentially expressed genes and proteins by day.

## Data Availability

All microarray datasets in this study are publicly available in the NCBI Gene Expression Omnibus database (GSE113478). We have a link for the review process, as below: Go to https://www.ncbi.nlm.nih.gov/geo/query/acc.cgi?acc=GSE113478, Enter token mholaekyvbsppyb into the box. The mass spectrometry proteomics data have been deposited to the ProteomeXchange Consortium via the PRIDE partner repository with the dataset identifier PXD037807. We have created a temporary user account for the review process, as below: Project Name: Longitudinal multi-omic changes in the transcriptome and proteome of peripheral blood cells after a 4 Gy total body radiation dose to Rhesus macaques. Project accession: PXD037807. Reviewer account details: Username: reviewer_pxd037807@ebi.ac.uk. Password: aXe7SFab.

## Abbreviations

TBI: Total body irradiation
mRNA: Messenger RNA
NHP: Non-human primate
PBMC: Peripheral blood mononuclear cell
H-ARS: Hematological acute radiation syndrome
MN/BN: Micronuclei per binucleate cell
CBMN: Cytokinesis-block micronucleus assay
DEG: Differentially expressed gene
WTLI: Whole thorax lung irradiation
