# Supplementary material for: Longitudinal multi-omic changes in the transcriptome and proteome of peripheral blood cells after a 4 Gy total body radiation dose to Rhesus macaques
Source: BMC Genomics. 2023 Mar 21;24:139. doi: 10.1186/s12864-023-09230-7 (PMC10031949; doi:10.1186/s12864-023-09230-7)

## Slide 1
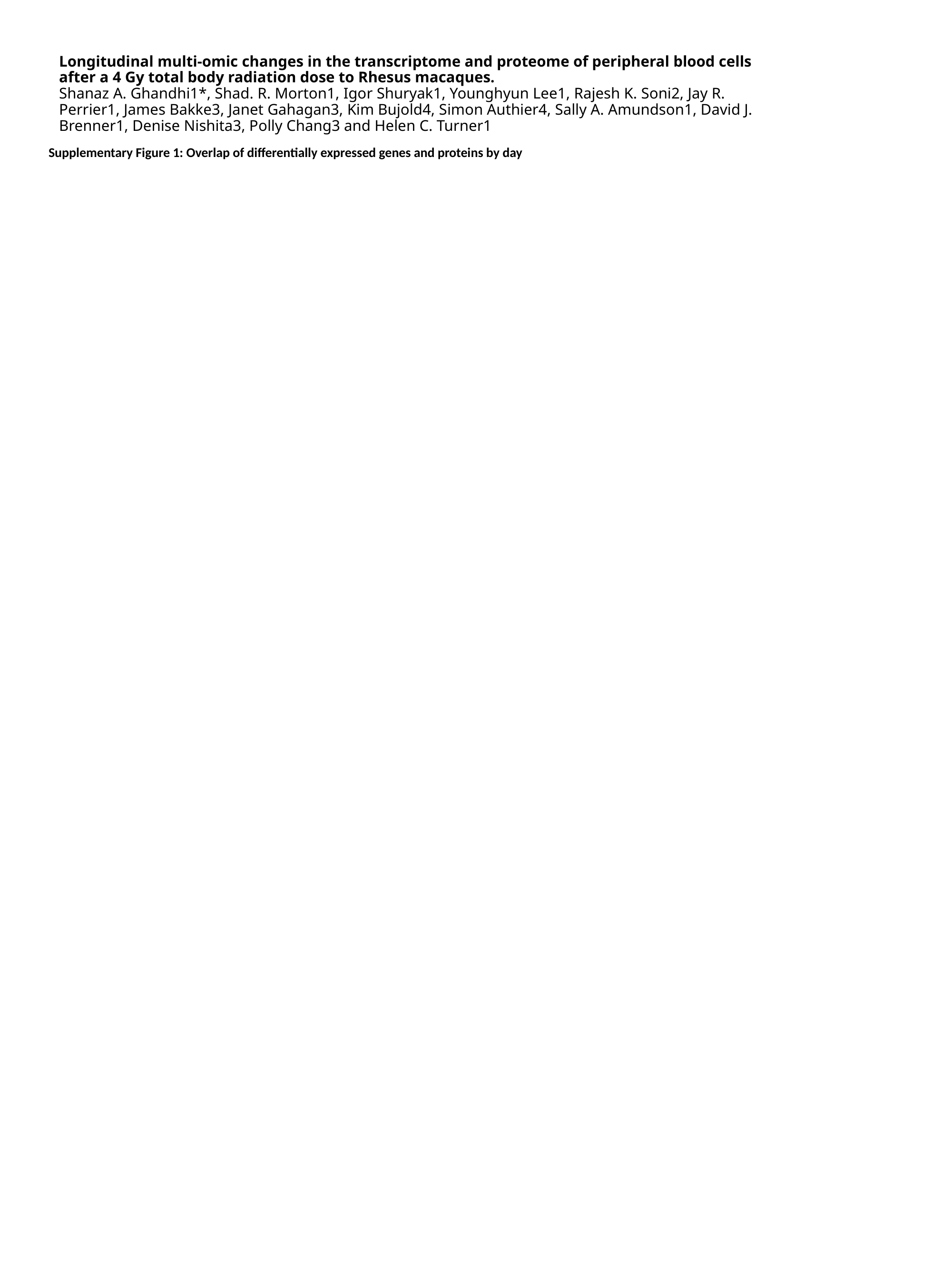

# Longitudinal multi-omic changes in the transcriptome and proteome of peripheral blood cells after a 4 Gy total body radiation dose to Rhesus macaques. Shanaz A. Ghandhi1*, Shad. R. Morton1, Igor Shuryak1, Younghyun Lee1, Rajesh K. Soni2, Jay R. Perrier1, James Bakke3, Janet Gahagan3, Kim Bujold4, Simon Authier4, Sally A. Amundson1, David J. Brenner1, Denise Nishita3, Polly Chang3 and Helen C. Turner1
Supplementary Figure 1: Overlap of differentially expressed genes and proteins by day

## Slide 2
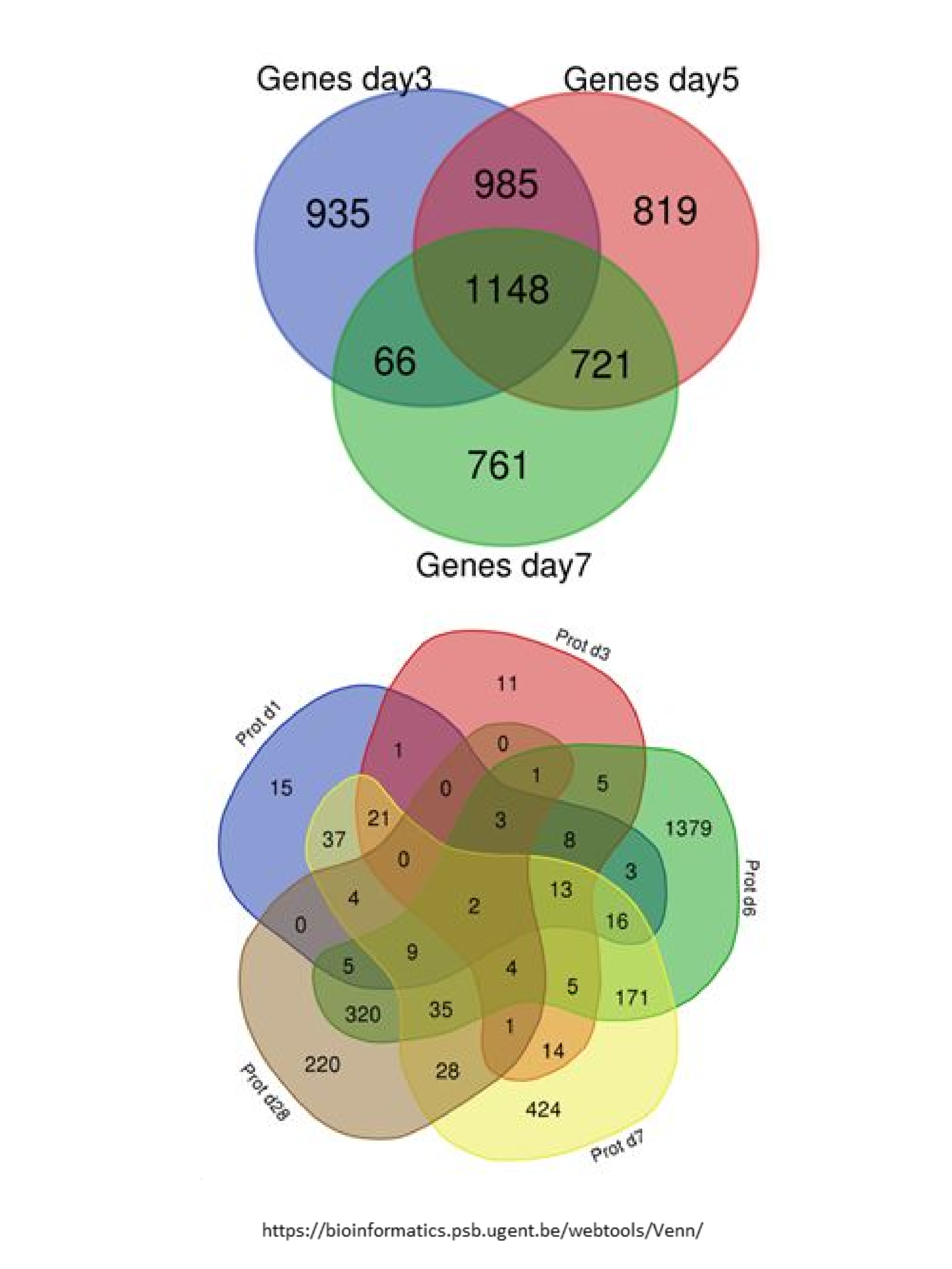

Supplement: Supplementary file 9 — Additional file 9: Supplementary Figure 1. Overlap of differentially expressed genes and proteins by day. [file 12864_2023_9230_MOESM9_ESM.pptx]
